# Sober by Act of Parliament

**Published:** 1894-10-27

**Authors:** 


### Studies in Applied Sociology.

"SOBER BY ACT OF PARLIAMENT."

Mr. Gladstone's pronouncement in favour of the Gothenburg system of licensing in preference to local option will undoubtedly rouse to new ardour the never-ceasing controversy on the best means of promoting temperance. The out-and-out teetotalers will doubtless condemn the aged statesman as an opportunist, for in their zeal for prohibition they refuse to see that restriction and guidance are at least steps in the right direction. At such a juncture, a book by Mr. Fred A. M'Kenzie, with the title which we have taken for our heading—"Sober by Act of Parliament" (London: Swan Sonnenschein and Co., price 3s. 6d.)—is a valuable addition to the literature of the subject. Mr. M'Kenzie details the action of the law to prevent drunkenness in various countries—prohibition in Maine, Kansas, and Iowa, with the recently-repealed State control in South Carolina, State monopoly in Switzerland, and the municipal system in vogue in Norway and Sweden.

Each system has a chronicle of success and failure. Prohibition has, on the whole, worked well in Maine, where the population is largely composed of native-born Americans, engaged in agriculture and fisheries. Maine is not to any great extent a manufacturing State, nor is it a State largely sought by immigrants. And in Maine prohibition has worked well, at least in the country districts. In the towns, although there is no town with more than forty thousand inhabitants, the tale is different. There, as elsewhere, the sale of alcohol as a beverage is forbidden, but the sale of alcohol for medicinal purposes is permitted, and, as Mr. M'Kenzie caustically notes, "Judging from the amount of whiskey sold as medicine in Portland, a considerable proportion of the inhabitants of the place must be chronic invalids."

In fact, prohibition cannot be forced on people. Where there is a strong public opinion in favour of temperance it can be enforced, at least openly, though illicit selling will never be wholly conquered; but where public sentiment does not back the law it becomes practically a dead letter. How often in the history of the world will it be necessary to repeat that the best of laws depend for efficacy on the spirit in which they are executed. Yet prohibition has its place, wherein it can do good work. In country districts the weak are at least saved from temptation; the whiskey-shop ceases to be the meeting-place of the community. Liquor is unattainable, except to adventurous young spirits, who feel it a point of honour to walk miles to get it, not because they love it, but because it is forbidden. On the whole the district will increase in temperance. But it is not in the country but in the towns that the drink fiend attains his worst results, and there prohibition conspicuously fails.

The alternative to prohibition, local or national, is local or national control of the liquor traffic, generally known to us as the Gothenburg and Swiss systems. In Switzerland the State is the sole liquor seller. The publicans are its paid servants, receiving a fixed salary, but no share of profits. They are there in fact, not to encourage drinking, but the reverse. The State is responsible for the quality of the spirit sold, thereby eliminating the incalculable mischief wrought by adulterated drink. Mr. M'Kenzie seems to regard this State monopoly as a moral failure. In this conclusion he differs from most English students of the subject. Financially, he says, the plan is a success; the State makes a profit. But this very fact frets the out-and-out prohibitionist, to whom alcohol in any form is simply "the accursed thing." The State as drink-seller makes drinking respectable, and those enter the State tavern who would not be seen in an ordinary public-house. But as a matter of fact, at least in Britain, we doubt if that argument has any weight with the majority of people. Those who drink to excess, those for whose sake prohibition or control is needed, are not the ones who stop to consider from whom they buy their liquor. And, physically, it is admitted that the State monopoly is useful. It at least gives good liquor to its customers. This, say the teetotalers, only enables them to drink more of it without injury. Perhaps so; but this is no such great misfortune. It is not drink but drunkenness that the State has to deal with, and the disease and crime that drunkenness brings in its train. If it can be proved that State control lessens the harm done by drink, even if it does not lessen the quantity drunk—which in Switzerland it has done, though not to any remarkable extent—the fact fully justifies the experiment.

The Gothenburg and Bergen systems resemble the Swiss in being intended to discourage rather than to prevent drinking; but the control is vested in a company which is invested with a monoply from the municipality. There is no doubt that in both cases the companies have used their powers honestly for the development of temperance, and therefore it is curious to see to what opposite extremes they have gone. In Bergen the drinking shop is a drink-shop and nothing else—a desolate unattractive place without even a seat. Whoso goes there goes only for his dram, and is not tempted to prolong his stay by company or good fellowship. In Gothenburg, on the other hand, the taverns partake more of the character of eating-houses. Food is to be obtained as well as drink, and the shops are bright, pleasant, and comfortable. So far as statistics go, the Gothenburg plan seems to answer best. When the company started there the amount drunk per head was 29 quarts; in 1891 it was only 14·3. Bergen can show nothing like this; but then Bergen had not such a thirsty population to start with. There the people drank only seven quarts per head annually at their worst; now they consume six. But this still leaves them much more temperate than their Swedish neighbours. It must be remembered, however, that in all Continental countries it is only the drinking of spirits that is fought against, beer and wine being looked upon as comparatively harmless, and their consumption tending to lessen the demand for more ardent liquors.

On the whole there is much to be said for the State management of the liquor traffic. Then only can public-houses be limited without injustice. At present to shut up half the licensed houses in a town serves only to increase the business at those remaining. Some publicans are ruined, and others are enriched. The State can see to it that it sells only pure spirits and beer, thereby preventing many of the worst effects of alcoholism and discouraging that raging thirst which results from "doctored" liquor. The State, receiving all the profit from the sale of liquor, could, by its paid tavern-keepers, discourage over-indulgence, yet receive such a revenue as would enable it to dispense with other more direct and burdensome taxes. These are practicable, attainable results. Can the best prospects of prohibition show more?

